# Feasibility of Hybriding Very High‐Power Short‐Duration and Ablation Index‐Guided Pulmonary Vein Isolation

**DOI:** 10.1002/joa3.70213

**Published:** 2025-10-24

**Authors:** Kyong Hee Lee, Atsuhiko Yagishita, Susumu Sakama, Iimura Kazuma, Takuji Kitazawa, Yuji Ikari, Koichiro Yoshioka

**Affiliations:** ^1^ Department of Cardiology Tokai University Isehara Japan

**Keywords:** Ablation Index‐guided ablation, atrial fibrillation, hybrid ablation, pulmonary vein isolation, very high‐power short‐duration ablation

## Abstract

**Introduction:**

A novel temperature‐controlled radiofrequency (RF) catheter enables pulmonary vein isolation (PVI) using very high‐power short‐duration (vHPSD) ablation, reducing esophageal injury risk but raising concerns about lesion durability in thicker atrial myocardium. This study aimed to assess the efficacy and safety of a hybrid approach that integrates conventional Ablation Index (AI)‐guided PVI with vHPSD ablation.

**Methods:**

This prospective, single‐center study enrolled 160 consecutive patients with atrial fibrillation (AF) between January 2023 and December 2023, who were allocated into two groups. Group 1 (*n* = 80) underwent conventional AI‐guided PVI using a 40 W setting, while Group 2 (*n* = 80) received a hybrid approach combining 90 and 50 W ablation with a temperature‐controlled RF catheter (QDOT Micro, Biosense Webster Inc., Diamond Bar, CA).

**Results:**

Group 2 demonstrated a significantly shorter duration for PVI compared to Group 1 (28 ± 11 min vs. 35 ± 10 min, *p* < 0.001), with similar rates of first pass isolation (86% vs. 89%, *p* = 0.63) and acute reconnection (10% vs. 5%, *p* = 0.23). Complication rates were comparable between the groups (1.3% vs. 1.3%, *p* = 1.00), with no cases of esophageal or phrenic nerve injury reported. Kaplan–Meier analysis showed no significant difference in freedom from AF at 1 year (84% vs. 83%, log‐rank *p* = 0.78).

**Conclusion:**

The integration of Ablation Index‐guided ablation with vHPSD ablation, utilizing a novel temperature‐controlled RF catheter, significantly reduces procedural duration while maintaining safety and efficacy comparable to conventional AI‐guided PVI.

## Graphical abstract

Compared to conventional Ablation Index (AI)‐guided ablation, the hybrid AI + very high‐power short‐duration (vHPSD) strategy significantly reduced pulmonary vein isolation time (28 ± 11 vs. 35 ± 10 min, *p* < 0.001), with comparable first‐pass isolation (86% vs. 89%, *p* = 0.63), acute reconnection (10% vs. 5%, *p* = 0.23), complication rates (1.3% vs. 1.3%), and 1‐year arrhythmia‐free survival (84% vs. 83%, log‐rank *p* = 0.78).

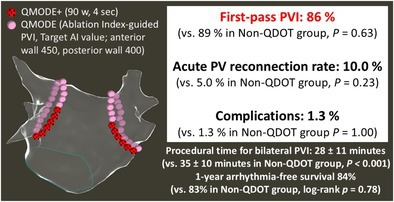

**Abbreviations**
AF: atrial fibrillation
AI: Ablation Index
AT: atrial tachycardia
FPI: first pass isolation
PFA: pulse field ablation
PV: pulmonary vein
PVI: pulmonary vein isolation
RF: radiofrequency
vHPSD: very high‐power short‐duration

## 1 Background

Pulmonary vein isolation (PVI) using radiofrequency (RF) ablation catheters has been a cornerstone in the catheter‐based treatment of atrial fibrillation (AF) [1]. Over the years, RF ablation technology has advanced significantly, transitioning from fixed power delivery to the adoption of the Ablation Index (AI, Biosense Webster Inc., Diamond Bar, CA) [2, 3]. AI integrates contact force, power, and time to predict lesion quality, enhancing the precision of ablation. However, conventional RF methods remain time‐intensive and are associated with risks of collateral damage [1].

To address these limitations, the QDOT Micro catheter (Biosense Webster) was developed, incorporating temperature‐controlled RF technology to improve efficiency, safety, and precision during PVI [4]. The QDOT Micro is equipped with integrated temperature sensors, enabling precise real‐time monitoring and control to ensure consistent lesion quality while minimizing the risk of overheating adjacent tissues. It delivers 90 W of RF energy for a maximum of 4 s per lesion, utilizing the very high‐power short‐duration (vHPSD) ablation technique. This approach creates lesions with large diameters and shallow depths, reducing the risk of esophageal injury compared to conventional RF catheters.

The Q‐FFICIENCY study demonstrated the catheter's ability to significantly reduce procedural times, with a median duration of 132 min. Additionally, the study highlighted the catheter's capability to create durable lesions with minimal esophageal injury [5]. However, concerns remain regarding the potential compromise in lesion durability in areas of thicker atrial myocardium. Therefore, this study aimed to evaluate the efficacy and safety of a hybrid approach integrating AI‐guided PVI with vHPSD ablation to address these limitations and optimize procedural outcomes.

## 2 Materials and Methods

### 2.1 Study Design and Population

This prospective, single‐center, non‐randomized cohort study enrolled 160 patients with AF between January 2023 and December 2023. Consecutive patients were assigned to two groups in chronological order: 80 patients underwent ablation using conventional 40 W settings guided by Ablation Index (Group 1), and 80 patients received a combination of AI‐guided and vHPSD ablation using the QDOT Micro catheter (Group 2). All patients provided written informed consent before undergoing the procedure. The study adhered to the ethical principles of the Declaration of Helsinki for human investigations and received approval from the Institutional Ethics Committee of Tokai University Hospital (approval no: 24R‐207).

### 2.2 Catheter Ablation Procedure

The absence of atrial thrombus was confirmed using transesophageal echocardiography or computed tomography prior to the procedure. All procedures were performed under conscious sedation using dexmedetomidine and midazolam with uninterrupted oral anticoagulation. An esophageal temperature probe was inserted into the esophagus to monitor the esophageal temperature during applications. When the temperature reached 39°C, the application was truncated, even if the target AI was not reached. Right femoral venous access was obtained, and a steerable dual‐decapolar electrode catheter (6F BeeAT; Japan Lifeline Co. Ltd., Tokyo, Japan) was positioned in the coronary sinus to enable simultaneous recording of the right atrium. After vascular sheath insertion, a 100 IU/kg heparin bolus was administered, and heparinized saline was infused to maintain an activated clotting time of 350–400 s.

**FIGURE 1:**
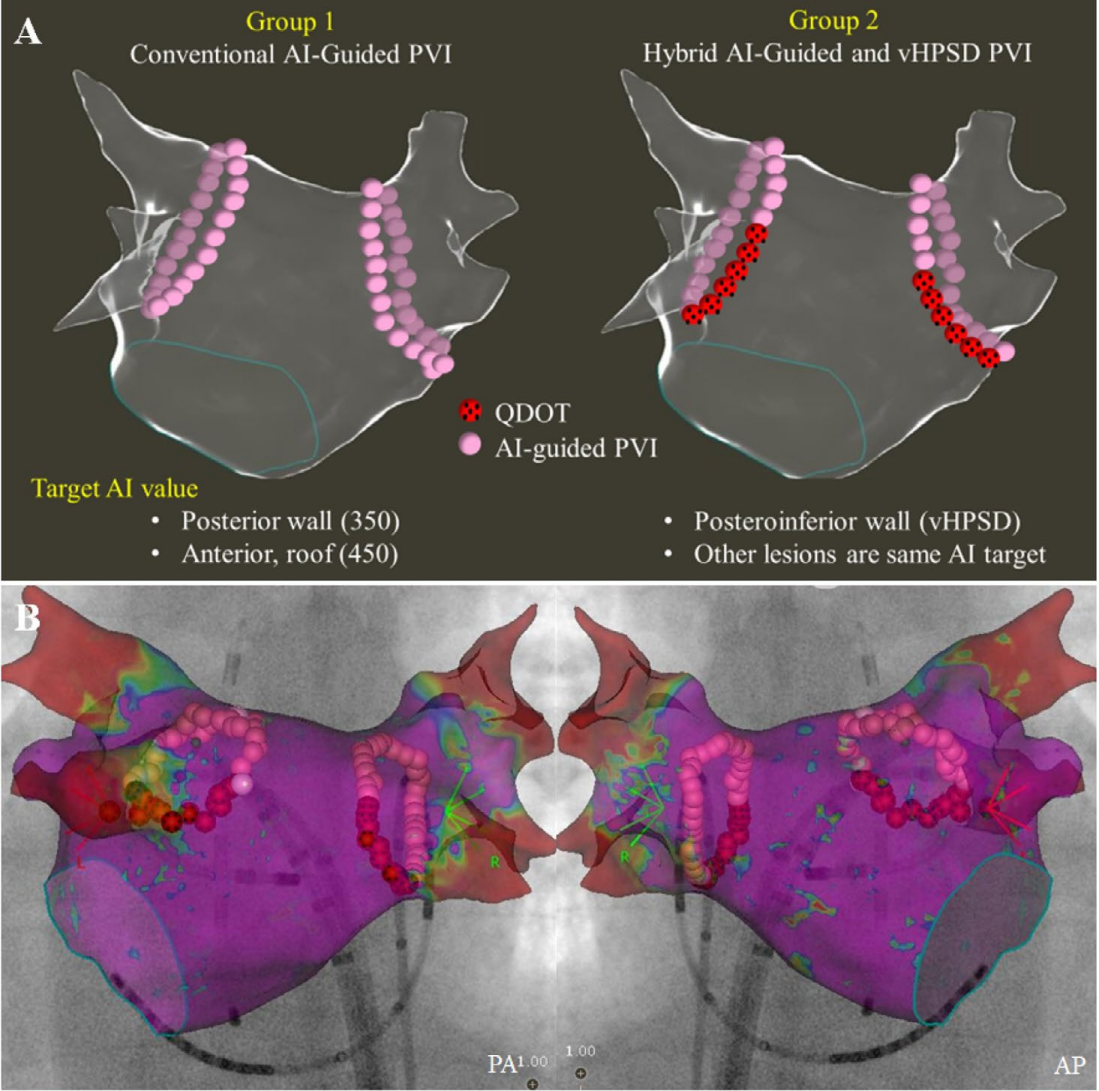
Pulmonary vein isolation strategy using Ablation Index and very high‐power and short‐duration approach. (A) Group 1 (Conventional AI‐Guided PVI): Ablation was performed with a power setting of 40 W, targeting an AI of 350 for the posterior wall, bottom, and roof, and 450 for the anterior wall. Group 2 (Hybrid AI‐Guided and vHPSD PVI): Ablation of the posterior inferior wall was performed using a 90 W power setting for 4 s in temperature‐controlled mode. The remaining regions were ablated at 50 W, with the same AI targets as in Group 1. AI, Ablation Index; AP anterior posterior view; PA, posterior anterior view; PVI, pulmonary vein isolation; RF, radiofrequency; vHPSD, very high‐power short‐duration. (B) Representative case of a hybrid AI‐guided and vHPSD PVI, demonstrating contiguous RF lesions with a lesion tag diameter of 4 mm and an interlesion distance of < 4 mm.

Bipolar electrograms were filtered between 30 and 500 Hz and displayed on a commercially available electrophysiological recording system (Nihon Kohden Corporation, Tokyo, Japan). Transseptal puncture was performed using an RF needle (Baylis Medical, Montreal, QC, Canada) and an 8‐F long sheath (SL‐0; SJM, Minneapolis, MN, USA). 3D electroanatomical mapping was conducted using a multielectrode mapping catheter (OctaRay Nav; Biosense Webster) and the CARTO3 version 7 ConfiDENSE Module (Biosense Webster). Before PVI, a detailed electroanatomical shell and voltage map of the left atrium were created during high right atrial pacing at a cycle length of 600 ms following cardioversion of atrial fibrillation. Following left atrium mapping, RF energy was delivered using either a contact force‐sensing ablation catheter, the Thermocool SmartTouch STSF (Biosense Webster) with power settings of 40 W for the anterior wall and 30 W for the posterior wall in Group 1, or the QDOT Micro catheter in Group 2, with a Stockert 70 RF generator (Biosense Webster) and a steerable VIZIGO sheath (Biosense Webster). Saline irrigation was maintained at 30 mL/min, and the temperature limit was set to 43°C. Lesion locations during RF applications were recorded using the VISITAG module with the following parameters: stability range of 2–3 mm, stability time of 3–5 s, force‐over‐time threshold of 25%, contact force range of 5–30 g, interlesion distance (ILD) of < 4 mm. During ablation, lesion tags were automatically generated using a tag size of 4 mm. The operator continuously monitored tag spacing in real time. In cases where visible gaps were identified between adjacent tags—suggesting an ILD exceeding 4 mm—additional ablation was applied to bridge the gap. This ensured the formation of a continuous lesion set with an intended ILD of < 4 mm (Figure 1).

#### 2.2.1 Group 1 (Conventional AI‐Guided PVI)

Ablation was performed with a power setting of 40 W, targeting an AI of 350 for the posterior wall, bottom, and roof, and 450 for the anterior wall.

#### 2.2.2 Group 2 (Hybrid AI‐Guided and vHPSD PVI)

Ablation of the posterior inferior wall was performed using a 90 W power setting for 4 s in temperature‐controlled mode (Figure 1). The remaining regions were ablated at 50 W, with the same AI targets as in Group 1.

### 2.3 Post‐Procedure Management

The patient underwent continuous electrocardiogram monitoring for 2 days after the procedure. The first outpatient clinic visit was 3 weeks after the procedure. Anti‐arrhythmic drugs were discontinued after the procedure, except for patients with early AF/atrial tachycardia (AT) recurrence, defined as an episode lasting ≥ 30 s occurring within 3 months post‐ablation (blanking period). Follow‐up visits comprised a clinical interview, electrocardiogram, and 24‐h Holter monitoring every 3, 6, and 12 months. After 12 months, the patients were observed every 6 months at our center. The last follow‐up entry dataset, which was based on the last office visit, was used to assess the long‐term freedom from recurrence. Long‐term freedom from AF/AT was based on the last visit using electrocardiogram or Holter electrocardiogram. Patients were maintained on oral anticoagulants for at least 3 months post‐procedure and thereafter according to the guideline recommendations [1].

### 2.4 Endpoints

The endpoints of the study were defined as follows: comparison of first‐pass isolation (FPI) rates, the number of RF applications required to achieve PVI, the time needed to complete PVI, the duration of RF energy application per lesion, acute reconnection rates for each pulmonary vein, procedure‐related complication rates, and freedom from AF and/or AT recurrence at 12 months between the two groups. Acute PV reconnection was defined as the reestablishment of electrical conduction between the PV and the left atrium following a 20‐min waiting period to assess for spontaneous reconnection after initial isolation, combined with the administration of isoproterenol at 5 μg/min for 10 min, in accordance with current consensus recommendations [1].

### 2.5 Statistical Analysis

Continuous variables are expressed as mean ± standard deviation or median and interquartile range (25th, 75th percentile). Continuous variables were compared using the unpaired *t*‐test or Mann–Whitney *U* test. The chi‐squared or Fisher's exact probability test was used to compare categorical variables. Survival curves were generated using Kaplan–Meier estimates, and time‐to‐event analyses were performed using the log‐rank test. A 2‐tailed *p*‐value < 0.05 indicated statistical significance. All statistical analyses were performed using JMP software (version 14.2).

## 3 Results

Table 1 summarizes the baseline characteristics of the 160 patients included in the study. The mean age was 68.3 ± 9.4 years, with 121 male patients (76%) and 101 patients (63%) diagnosed with persistent AF. The median CHADS_2_ score was 1. The duration of AF was significantly longer in Group 1 compared to Group 2 (0.5 vs. 0.3 years, *p* = 0.039), while the use of diuretics was lower in Group 1 (15% vs. 29%, *p* = 0.039). No significant differences were observed in other baseline clinical parameters between the groups.

**TABLE 1:** Baseline characteristics.

	All patients (*n* = 160)	Group 1 (Conventional AI‐Guided PVI) (*n* = 80)	Group 2 (Hybrid AI‐Guided and vHPSD PVI) (*n* = 80)	*p*
Age (year)	68.3 ± 9.4	68.5 ± 9.7	68.1 ± 9.2	0.78
Male (%)	121 (76)	63 (79)	58 (73)	0.36
Body mass index (kg/m^2^)	24.0 ± 4.1	24.1 ± 4.1	23.9 ± 4.2	0.66
Persistent AF (%)	101 (63)	46 (58)	55 (69)	0.14
Duration of AF (IQR), years	0.4 (0.3–1.3)	0.5 (0.3–2.3)	0.3 (0.3–0.9)	0.039
Persistent duration (IQR), years	0.2 (0–0.4)	0.2 (0–0.5)	0.3 (0–0.4)	0.18
Anti‐arrhythmic drug use (%)	32 (20)	19 (24)	13 (16)	0.23
Hypertension (%)	96 (60)	46 (58)	50 (63)	0.52
Diabetes mellitus (%)	33 (21)	20 (25)	13 (16)	0.17
Heart failure (%)	45 (28)	18 (23)	27 (34)	0.11
Cerebral infarction (%)	9 (6)	6 (8)	3 (4)	0.3
CHADS2 score (IQR)	1 (1–2)	1 (1–2)	1 (1–2)	0.63
CHA2DSVACS score (IQR)	2 (1–3)	2 (1–4)	2 (1–3)	0.5
Diuretics (%)	35 (22)	12 (15)	23 (29)	0.034
Beta blocker (%)	84 (53)	44 (55)	40 (50)	0.53
Digoxin (%)	3 (2)	0 (0)	3 (4)	0.04
ACEi (%)	11 (7)	5 (6)	6 (8)	0.75
ARB/ARNI (%)	72 (45)	33 (41)	39 (49)	0.34
Left atrial diameter (mm)	40.5 ± 5.2	40.6 ± 5.1	40.3 ± 5.3	0.74
Ejection fraction (%)	60.1 ± 11.8	61.9 ± 10.9	58.3 ± 12.5	0.052
Creatinine (IQR), mg/dL	1.00 (0.83–1.15)	1.00 (0.84–1.15)	1.00 (0.81–1.15)	0.94
eGFR (IQR), mL/min/1.73m^2^	55.0 (48.0–66.0)	55.0 (48.5–64.0)	55.0 (46.3–67.8)	0.98
HbA1c (IQR), %	6.0 (5.8–6.4)	6.0 (5.7–6.3)	6.1 (5.8–6.4)	0.14
BNP (IQR), pg/mL	111.4 (51.7–219.8)	88.5 (49.8–195.2)	134.3 (54.9–249.1)	0.09

Abbreviations: ACEi, Angiotensin‐converting‐enzyme inhibitor; AF, Atrial fibrillation; AI, Ablation Index; ARB, Angiotensin receptor blocker; ARNI, Angiotensin receptor/neprilysin inhibitor; BNP, B‐type natriuretic peptide; eGFR, Estimated glomerular filtration rate; PVI, pulmonary vein isolation; vHPSD, very high‐power short‐duration.

FPI was achieved in 86% of both pulmonary veins in Group 1, which was comparable to Group 2 (89%, *p* = 0.63). In Group 2, FPI was achieved in 97% of left PVs and 90% of right PVs, with an acute reconnection rate of 4% per PV. The mean time required to complete PVI was 32 ± 11 min, with 15 ± 7 min for the left PVs and 17 ± 6 min for the right PVs. The mean number of RF applications necessary for PVI was 39 ± 14 for the left PVs and 46 ± 14 for the right PVs. The mean RF energy application duration per lesion was 23 ± 4 s for the left PVs and 22 ± 4 s for the right PVs. Procedure‐related complications occurred in two patients (1.3%).

**FIGURE 2:**
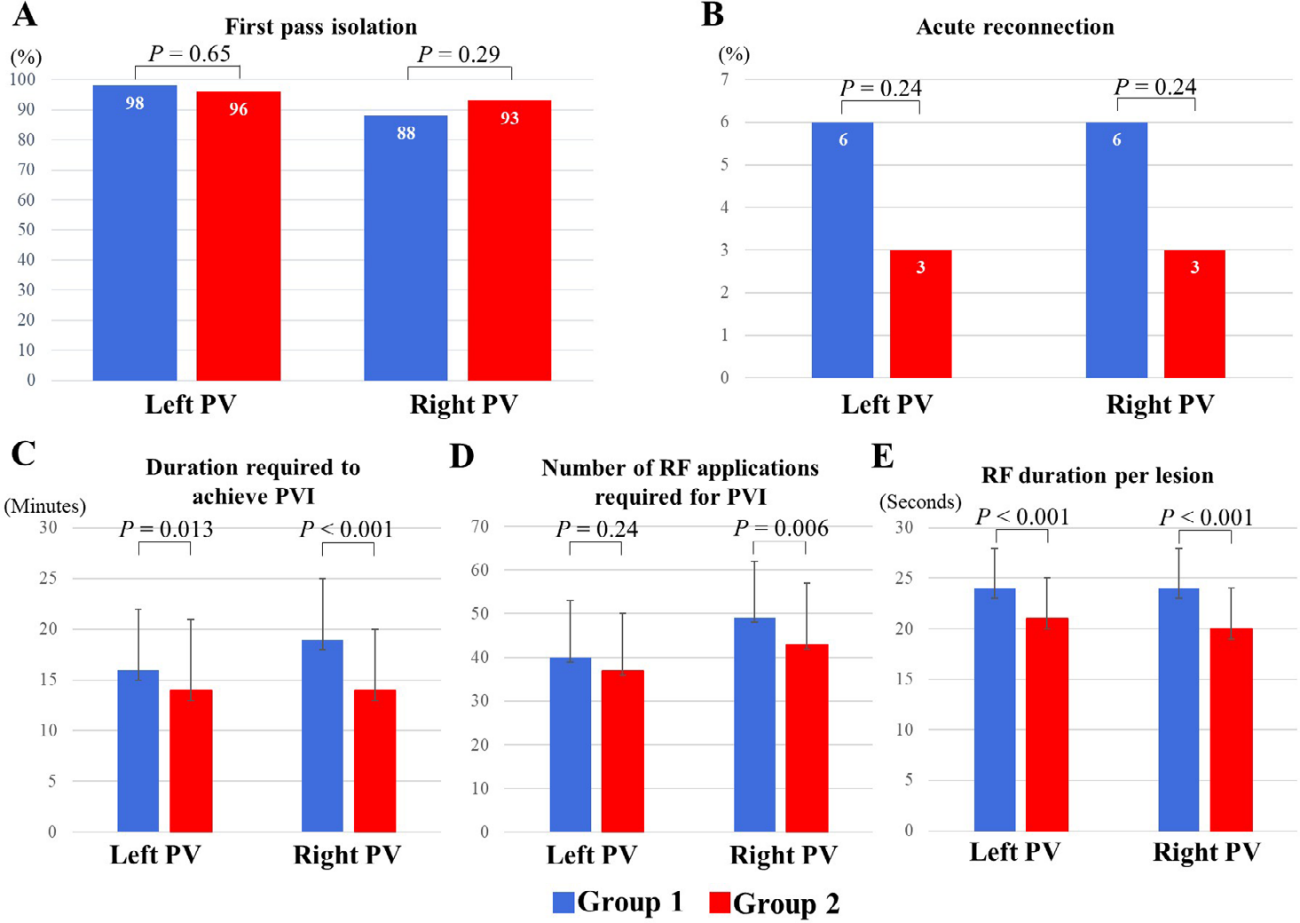
Comparison of procedural parameters between the two groups. (A) First pass isolation rates were comparable between the groups (98% vs. 96%, *p* = 0.65 for the left PVs; 88% vs. 93%, *p* = 0.29 for the right PVs). (B) Acute reconnection rates showed no significant difference for both the left PVs (6% vs. 3%, *p* = 0.24) and the right PVs (6% vs. 3%, *p* = 0.24). (C) The time required to achieve PVI was significantly shorter in Group 2 for both the left PVs (16 ± 6 vs. 14 ± 7 min, *p* = 0.013) and the right PVs (19 ± 6 vs. 14 ± 6 min, *p* = 0.001). (D) Group 2 required fewer RF applications for the right PVs compared to Group 1 (49 ± 13 vs. 43 ± 14, *p* = 0.006). (E) The RF energy application duration per lesion was significantly shorter in Group 2 for both the left PVs (24 ± 4 vs. 21 ± 4 s, *p* < 0.001) and the right PVs (24 ± 4 vs. 20 ± 4 s, *p* < 0.001). PV, pulmonary vein; PVI, pulmonary vein isolation; RF, radiofrequency.

**FIGURE 3:**
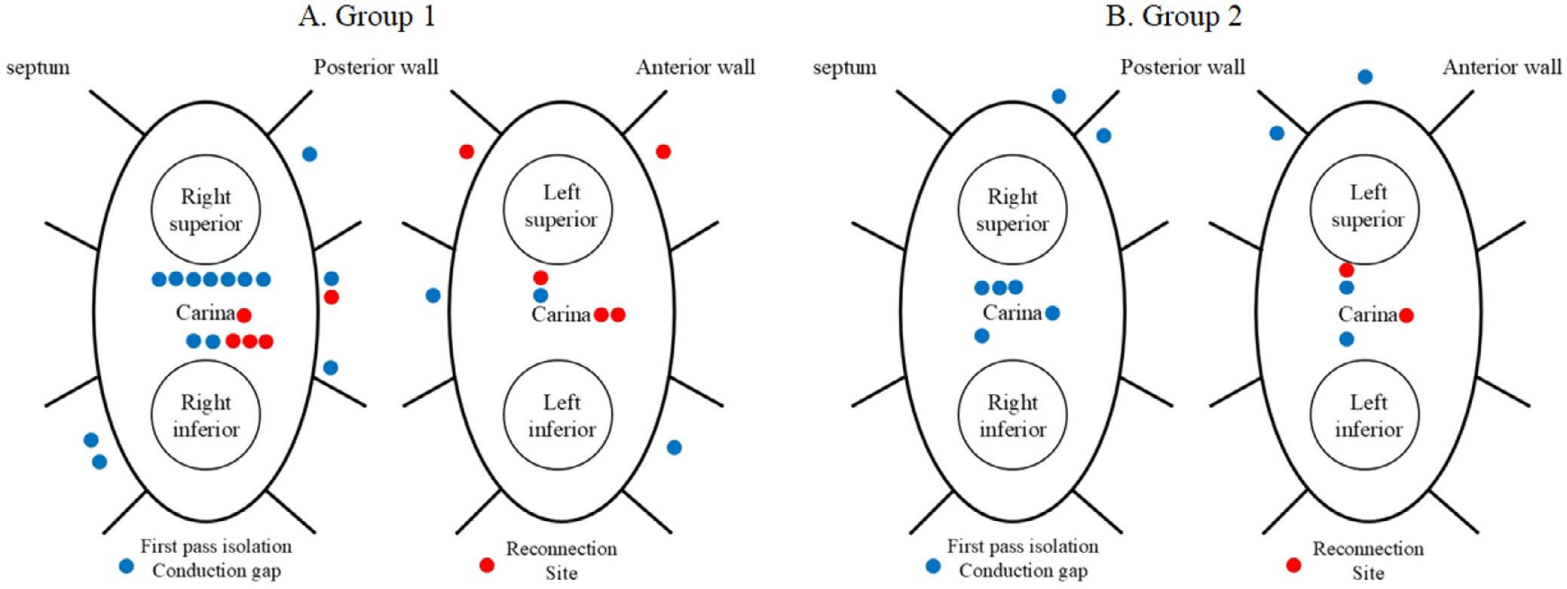
Distribution of conduction gaps and sites of reconnection in both groups. Schematic representation of the distribution of conduction gaps and reconnection sites following PVI. Panel A shows the locations in Group 1 (conventional Ablation Index‐guided PVI), and Panel B shows those in Group 2 (hybrid PVI using AI‐guided and vHPSD ablation). Blue dots indicate conduction gaps identified after first‐pass isolation, and red dots represent sites of reconnection observed during the procedure. The carina region and anatomical orientation (septum, posterior wall, anterior wall) are labeled for reference. Notably, conduction gaps were frequently observed in the carina region in both groups.

Figure 2 illustrates the procedural parameter comparisons between the two groups. Although FPI rates were comparable (98% vs. 96%, *p* = 0.65 for the left PVs; 88% vs. 93%, *p* = 0.29 for the right PVs), the time required to achieve PVI was significantly shorter in Group 2 for both the left PVs (16 ± 6 vs. 14 ± 7 min, *p* = 0.013) and the right PVs (19 ± 6 vs. 14 ± 6 min, *p* = 0.001). Group 2 also required fewer RF applications for the right PVs (49 ± 13 vs. 43 ± 14, *p* = 0.006), and the RF energy application duration per lesion was shorter for both the left PVs (24 ± 4 vs. 21 ± 4 s, *p* < 0.001) and the right PVs (24 ± 4 vs. 20 ± 4 s, *p* < 0.001). Despite these procedural differences, acute reconnection rates remained similar between the groups for both the left PVs (6% vs. 3%, *p* = 0.24) and the right PVs (6% vs. 3%, *p* = 0.24). The most common conduction gap was carina in both groups, and the others were sporadic distribution (Figure 3). The incidence of complications was equivalent between the groups (1.3% vs. 1.3%, *p* = 1.00), with one case of cardiac tamponade in Group 1 and one case of pericarditis in Group 2. No esophageal or phrenic nerve injuries were reported in either group.

**FIGURE 4:**
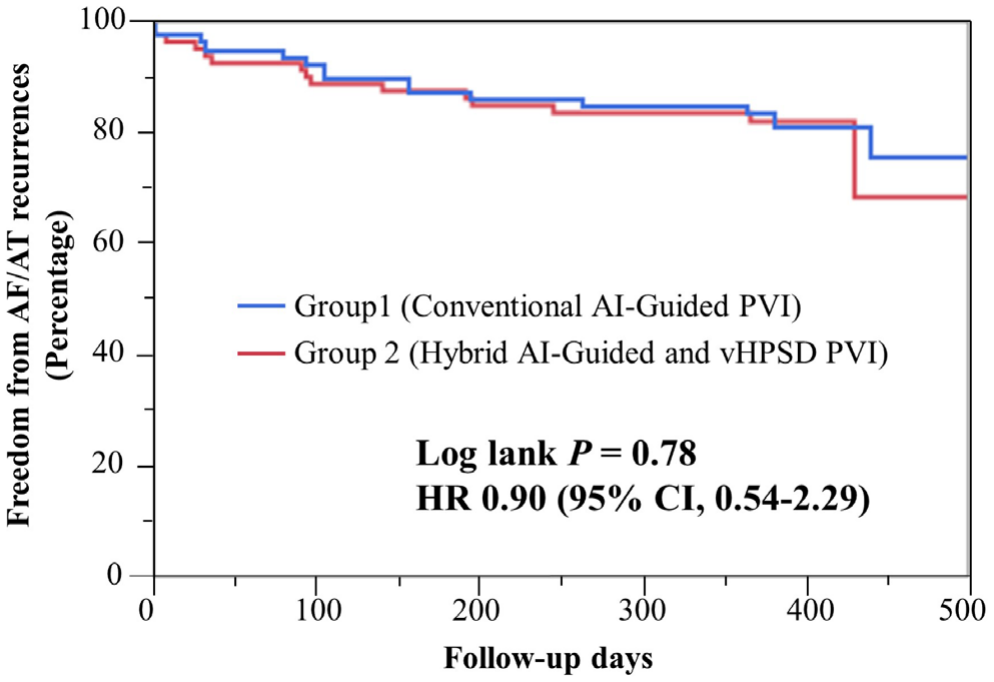
AF/AT recurrence during long‐term follow‐up. At 12 months of follow‐up, 133 out of 160 patients (17%) were free from AF/AT recurrence, including 67 patients in Group 1 (84%) and 66 patients in Group 2 (83%). Freedom from AF recurrence was comparable between the two groups, with no statistically significant difference (log‐rank *p* = 0.78; HR: 0.90; 95% CI: 0.54–2.29). AF, atrial fibrillation; AT, atrial tachycardia.

At 12 months of follow‐up, 133 out of 160 patients (17%) were free from AF/AT recurrence, including 67 patients in Group 1 (84%) and 66 patients in Group 2 (83%). Freedom from AF recurrence was comparable between the two groups, with no statistically significant difference (log‐rank *p* = 0.78; HR: 0.90; 95% CI: 0.54–2.29, Figure 4).

## 4 Discussion

This study aimed to determine the efficacy and safety of a hybrid approach integrating AI‐guided PVI with vHPSD ablation. Our main findings were as follows: (1) the overall time required to achieve PVI was significantly shorter in Group 2 for both PVs, (2) Group 2 also required fewer RF applications for the right PVs and had shorter RF energy application durations per lesion for both PVs, (3) despite these procedural advantages, first pass isolation rates, acute reconnection rates, and complication rates were comparable, and (4) at 12 months of follow‐up, freedom from AF recurrence was similar between the groups.

Conventional RF ablation methods are time‐intensive and associated with risks of collateral damage [1]. To address these limitations, the QDOT Micro catheter was developed, featuring very high‐power ablation and precise temperature control [2, 3]. This catheter reduces procedure times while maintaining safety. However, previous studies comparing vHPSD and standard RF ablation have shown that vHPSD lesions tend to have larger diameters but shallower depths, potentially increasing the risk of acute reconnections in regions with thicker atrial walls [6]. As previously noted in studies such as the QDOT‐by‐left atrial wall thickness trial, vHPSD lesions tend to be broader but shallower compared to conventional RF ablation, potentially increasing the risk of acute reconnections in areas with thicker atrial walls. This limitation has prompted interest in tailoring ablation strategies based on left atrial wall thickness, as demonstrated by left atrial wall thickness‐guided approaches that personalize energy delivery to achieve transmural lesions [6]. However, widespread implementation of left atrial wall thickness‐guided ablation is challenging due to the need for advanced imaging modalities and increased procedural complexity. To simplify this approach, our study combined AI‐guided ablation with vHPSD techniques, leveraging established knowledge of anatomical left atrial wall thickness and lesion depth relationships. The left atrium wall is composed of an intermingled series of muscles, chief of these being the interatrial band and the septoatrial bundle. On average, the left atrium wall is more uniform and thicker than the right atrial wall [7]. In a study by Sien Yen Ho et al., the anterior wall thickness of the left atrium was reported as 3.3 ± 1.2 mm, while the posterior wall measured 4.1 ± 0.7 mm, gradually thinning toward the PV orifices. At the veno‐atrial junction, the thickness was approximately 1–1.5 mm. Similarly, atrial wall thickness around the superior PV ranged from 1.5 to 4.5 mm, and from 0.5 to 3.5 mm around the inferior PV [8, 9]. It should be noted, however, that the measurements reported by Ho et al. were obtained from autopsied normal hearts. In contrast, other anatomical and imaging studies involving patients with atrial fibrillation have consistently shown that the posterior left atrial wall is typically thinner than other regions, with reported thicknesses ranging from 2.3 to 2.9 mm [10]. This discrepancy likely reflects atrial remodeling associated with AF, including wall thinning and fibrosis. Based on these anatomical insights, we applied 90 W for 4 s specifically in thinner regions—such as the bilateral posterior‐inferior walls—while conventional AI‐guided parameters were used for the remaining areas. Additional studies, such as that by Felix Bouier et al., have demonstrated that a 13‐s ablation at 50 W achieved an AI of ~500 and a lesion depth of 4.7 ± 0.6 mm [2], supporting the correlation between lesion depth and AI reported by Mori et al. [11]. Based on these findings, this study demonstrated the clinical feasibility of a hybrid approach combining AI‐guided and vHPSD ablation using the QDOT Micro catheter. This strategy significantly reduced procedural times while maintaining comparable long‐term efficacy and safety, providing a simplified and effective alternative to conventional AI‐guided ablation without compromising patient outcomes. Despite the use of this strategy, reconnection at the carina was frequently observed, consistent with previous reports [12]. This may be attributed to different anatomical challenges: in the right pulmonary vein, epicardial connections are likely to be involved, whereas in the left pulmonary vein, catheter instability during ablation or increased local atrial wall thickness may contribute to incomplete lesion formation [13].

In this study, some differences were observed compared to previous reports. In the QDOT FAST trial, PVI was achieved in 79% of patients using only the vHPSD setting, while in the SHORT AF trial, the rate of PVI was 79% with HPSD and 76% with standard power settings [14, 15]. In the peQasus multicentre study, which evaluated a hybrid approach, the FPI rate ranged from 73.5% to 79.7% [16]. These rates were slightly lower than those observed in our study. One possible explanation for this difference is the variation in ILD settings. In the peQasus multicentre study, the ILD in the hybrid group was 5–6 mm, which was associated with a lower number of applications compared to our protocol (ILD < 4 mm), highlighting the importance of tighter lesion spacing to improve FPI rates [16]. To the best of our knowledge, this is the first study to demonstrate the superiority of a hybrid ablation strategy using a tight ILD distance (< 4 mm) over conventional AI‐guided ablation.

Recent advancements in pulsed field ablation have transformed AF treatment by utilizing irreversible electroporation to selectively ablate cardiac myocytes while preserving adjacent structures, such as the esophagus and phrenic nerve [17, 18]. While PFA is rapidly gaining popularity due to its tissue selectivity and safety profile, limitations remain—particularly in creating precise and durable lesions in complex anatomical regions or when treating non‐PV triggers. Therefore, conventional RF ablation, including hybrid strategies such as the one evaluated in this study, continues to play a central role in AF ablation procedures. As noted in a recent review [19], PFA offers promising advantages but also presents challenges that warrant further evaluation in diverse clinical scenarios. Furthermore, while there have been reports of PFA applications for cavotricuspid isthmus or mitral isthmus ablation in cases of concomitant AT or flutter [20, 21], the validity and long‐term durability of these procedures remain uncertain. As a result, we believe that RF ablation remains a cornerstone of PVI, particularly in scenarios requiring precise lesion formation or the treatment of concomitant AT or flutter, where the advantages of RF technology are well‐established.

## 5 Limitations

There were some limitations in this study. First, while our approach simplifies energy delivery based on general anatomical knowledge, it does not account for patient‐specific variations in left atrial wall thickness, which may affect clinical outcomes. Second, longer‐term follow‐up beyond 12 months is needed to confirm the durability of lesions created using this strategy. Further research is essential to refine patient‐specific approaches and evaluate the long‐term outcomes of this technique. Third, although planned prospectively, the study was not powered as a randomized controlled trial and no formal a priori power calculation was performed. Consequently, the study may be underpowered to detect modest differences in efficacy or rare adverse events, and the findings should be interpreted as exploratory. Larger, adequately powered randomized studies are required to confirm these preliminary observations. Fourth, because this study was not a randomized controlled trial, baseline imbalances—such as the use of diuretics, digoxin, and the duration from diagnosis to ablation—were noted. These differences may have influenced the study outcomes. Finally, although the periprocedural complication rate was low, PV stenosis during long‐term follow‐up was not systematically assessed in this study, potentially underestimating the incidence of silent PV stenosis.

## 6 Conclusion

This study demonstrated that the combined 90 W/50 W ablation strategy using the QDOT Micro catheter significantly reduces procedural time compared to conventional AI‐guided ablation, while maintaining comparable long‐term efficacy and safety without compromising patient outcomes.

## Ethics Statement

This study was conducted in accordance with the principles of the Declaration of Helsinki regarding investigations in humans and was approved by the Institutional Ethics Committee of Tokai University Hospital (approval no. 24R‐207).

## Conflicts of Interest

Dr. Yagishita received honoraria from Medtronic, Johnson & Johnson, and Abbott outside the submitted work. The other authors declare no conflicts of interest.
